# Has aging ever been considered healthy?

**DOI:** 10.3389/fgene.2015.00202

**Published:** 2015-06-05

**Authors:** Ilia Stambler

**Affiliations:** Department of Science, Technology and Society, Bar-Ilan UniversityRamat Gan, Israel

**Keywords:** history of medicine, healthy aging, anti-aging, longevity research, aging-related diseases, life extension

The current research topic inquires: “Should we treat aging as a disease?” Yet, in this inquiry, the question “Can aging be considered a disease?” is secondary, while the more primary question really must be “Is aging treatable?” Paradoxically, the answer given to the second question largely determines the answer to the first. The perceived unchangeable, and hence untreatable, nature of aging is the root cause for many subsequent rationalizations, even to the point of claiming the desirability of aging-derived suffering and death. This is a well recognized psychological phenomenon sometimes referred to as “apologism” (Gruman, 1966) or even “deathism,” a ramification of the “sour grapes syndrome,” vilifying something that we think we cannot attain, while accepting as “good” or “healthy” something that we believe is inevitable for us (such as degenerative aging). Yet, I argue that, historically, medical tradition has always recognized the morbid character of aging and endeavored to fight it. The rationalizations of aging as “natural,” “justified,” or “healthy” could never entirely prevail.

An example of this recognition can be found as early as “On Old Age” (*De Senectute*) by Cicero (106-43 BCE). It was written at a time when average life-expectancy reached about 30 years, when even survival to old age was a rare occasion. Consistently with the “apologetic” tradition, *De Senectute* presents a picture of old age that not only endeavors to “wipe away all the disagreeables of old age” but even to present it as “luxurious and delightful too” (Cicero, 1900). Cicero even directly stated the reason to consider aging and death from aging as attractive, namely, the impossibility to oppose them: “But those who look for all happiness from within can never think anything bad which nature makes inevitable.” Yet, Cicero's common sense would not allow him to completely block out of mind the decay and suffering caused by old age, neither to completely deny the human ability to greatly retard this decay through proper care of the body and mind. Hence alongside the call for ultimate philosophical resignation with aging and death, there is still a practical call to “fight” the infirmities and feebleness produced by aging:

“We must stand up against old age and make up for its drawbacks by taking pains. We must fight it as we should an illness. We must look after our health, use moderate exercise, take just enough food and drink to recruit, but not to overload, our strength. Nor is it the body alone that must be supported, but the intellect and soul much more.”

This is the practical stance that prevailed in the literature of “gerocomia” or “gerocomica” (from the Greek “treatment of old age”), from the Classical through the Medieval well into the Modern times, from the writings of Galen (c. 129-217 AD), through Gabriele Zerbi (1445-1505) and Luigi Cornaro (1467-1566) to Leonardus Lessius (1554-1623), Johann Heinrich Cohausen (1665-1750) and other hygienists (Stambler, 2014a). In this type of writings, there is often an ultimate philosophical resignation to aging and mortality, yet there is also a practical desire to postpone them for as much as possible.

It can be observed that with the beginning of the modern period, with human advancement, with expanding geographical and technological conquests, and with the strengthening of the scientific worldview, the proactive militant attitude toward aging appears to move further and further to the foreground, while resignation and rationalizations retreat. A striking example of this growing proactive attitude, in the early modern period, can be found in the writings of the seventeenth century British physician John Smith, who urged (Smith, 1666):

“Let none give over their patients when they come overburdened with the infirmities of Age, as though they were altogether incapable of having any good done unto them. Those that are negligent toward their Ancient Friends, are very near of kin to those inhuman Barbarians and Americans, who both kill and devour them.”

This could be seen as setting the goal for the treatment of aging-related ill health for the modern period. Since then, there have been many examples continuing and developing thlis attitude.

Treatments for aging-related health damage have been sought, among others, by Christoph Wilhelm Hufeland (1762-1836), the renowned German hygienist, who coined the term “macrobiotics”—the science of life prolongation. According to him, reaching a lifespan of 200 years may be feasible and “natural” for humans. He perceived “the old age” as “the grand enemy of life,” and as a diagnosable condition, like a disease, not so much dependent on the actual passage of years, as on the internal disarray of the organism: “and one may see very old people of 30 or 40, who have every symptom of extreme age, such as stiffness and aridity, weakness, gray hair, ossified cartilages, &c.” Even though, according to Hufeland, aging can be generally seen as a morbid condition, his objective is actually more complex than a simplistic attempt to eradicate aging (or rejuvenate the body) at once. Though Hufeland saw the degeneration brought on by aging, his ultimate goal was not rejuvenation for the sake of rejuvenation, but “macrobiotics”—the prolongation of life. And paradoxically, “rejuvenation,” an immediate improvement of function, can become an enemy of life prolongation:

“Old age, though the natural consequence of living, and the commencement of death, can itself, on the other hand, be a means for prolonging our existence. It does not, however, increase the power to live, but it retards its being exhausted …Man, during the period of old age, has a much smaller provision of vital power, and a much less capacity for restoration. If he lived with the same activity and vigor as before, this provision would be much sooner exhausted, and death would soon be the consequence. Now the character of age lessens the natural irritability and sensibility of the body, by which the effects of internal as well as external irritation, and consequently the exertion and wasting of the powers, are also lessened…”

In other (perhaps more modern) terms, any treatment of old age should consider the aging organism and the aging process as a whole. Any attempt to artificially strengthen some faculty, at the disregard of the general adaptation and available resources of the entire aging system, can further advance the disarray and bring about death sooner. This is a lesson by Hufeland that may be well heeded by some contemporary reductionist “anti-aging” attempts. Still, despite the caution and recognition of complexity, the necessity and possibility of “treatment” of old age as a disease-like, deteriorative condition is recognized. Caution and thoughtfulness just need to become parts of its “proper treatment” [Hufeland, 1867 (1796), “Old Age. Proper Treatment of It”].

Later on, the fact that aging can be treated was further recognized by pioneers of modern medicine. The pliability of aging was stated, among others, by Charles-Édouard Brown-Séquard (1817-1894), one of the founders of modern endocrinology, president of the French Biological Society and Claude Bernard's pupil and successor at Collège de France. In the widely publicized presentation to the French Biological Society of June 1, 1889, entitled “Effects in man of subcutaneous injections of freshly prepared liquid from guinea pig and dog testes” (Brown-Séquard, 1889a), he announced his first attempts at hormone replacement therapy for rejuvenation. This presentation, in fact, introduced longevity and rejuvenation research as an integral part of scientific discourse, and established the field of therapeutic endocrinology. Brown-Séquard posited the treatable and thus disease-like nature of aging explicitly (Brown-Séquard, 1889b):

“They show great ignorance who maintain that it is impossible in old men to reverse their organic state so that they resemble that of an earlier age, especially since the organic changes resulting from better nutrition are possible at all ages…Critics of my ideas have said that it is well known that senile degeneration and wasting present insurmountable obstacles, especially return of neural center function both in the sensory and the motor apparatus. A study of the excellent work of Charcot on Aging (Studies of Diseases of Old Men, Paris, 1868) and a number of other works show that nothing about senility is constant nor absolutely characterized. …If the degenerations, if the senile alterations are diseases, a day will come when it will be possible to cure them.”

The undaunted fighting attitude toward “degeneration and wasting” may have been an indispensable part of the spiritual and intellectual drive that enabled becoming a pioneer of medical science in the first place.

Another pioneer of modern medical science, as well as a fighter against aging, was Elie (Ilya Ilyich) Metchnikoff (May 15, 1845—July 15, 1916). Metchnikoff is of course known as a groundbreaking immunologist, a vice director of the Pasteur Institute in Paris, and the Nobel Laureate in Physiology or Medicine of 1908 for the discovery of phagocytosis (a major contribution to the cellular theory of immunity). Yet, he may also be well credited as “the father” of gerontology—the disciplinary term he coined in 1903 in his book *Etudes On the Nature of Man*. Metchnikoff argued that extreme longevity can be achieved through the progress of medical science, requiring a massive collective effort. Metchnikoff believed that it is our duty as conscious human beings to fight death, the main disharmony and evil of nature. He strongly emphasized that each death has an identifiable and treatable cause and in this sense every death is “violent” and not “natural.” The fact that everyone must succumb to it does not make it right or even acceptable. Death from aging is no exception [Metchnikoff, 1961 (1903)]:

“It has been long noted that aging is very similar to disease. Therefore, it is not surprising that human beings feel a strong aversion to aging. …Undoubtedly, it is a mistake to consider aging as a physiological phenomenon. It makes as much sense to accept aging as a normal phenomenon, because everybody ages, as it makes sense to accept childbirth pain as normal, because only very few women are spared it. In both cases, we deal, of course, with pathological and not with purely physiological phenomena. Inasmuch as people endeavor to mitigate or eliminate the pains of a woman in labor, it is as natural to endeavor to eliminate the evils brought by aging. However, while during childbirth pains, it is enough to apply an anesthetic, aging is a chronic evil against which it is much more difficult to find a cure.”

Though, Metchnikoff hypothesized that there might be a point in human life when death will occur naturally, as it is programmed (at about 150 years), we are yet very far from that limit. For the present stage of human condition, the imperative to combat aging and prolong life were posited by Metchnikoff unequivocally. This seems to be a stance befitting a groundbreaking medical scientist. And indeed, during the fight with aging, novel therapies were born, applicable not just to general degeneration but also to specific diseases, including hormone replacement therapies introduced by Brown-Séquard during the study of rejuvenation, or systemic adjuvant immunotherapy and probiotic diets pioneered by Metchnikoff during his study and combat of aging (Stambler, 2014b).

Thus, I argue, acknowledging the possibility of successful intervention into the aging process, in other words treating aging as a curable disease, has been a long and highly respected tradition of biomedical thought. A comprehensive historical overview of the debates regarding the possibility to intervene into or treat aging, from the earlier date until our times, would go far beyond the scope of the present work. An attempt at such a comprehensive history is presented elsewhere (Stambler, 2014a). It may just be observed that the proactive attitudes, aimed to ameliorate degenerative aging, tend to intensify thanks to the advancement of technological capabilities. Presently, the list of supporters of the cause of “curing aging” grows rapidly. The reason for this increase may be objective and tectonic. The world is rapidly aging, threatening grave consequences for the global society, in particular economy, which forces the society to seek solutions. On the other hand, biomedical science and technology are developing rapidly as well, increasing the feasibility of intervention and fostering our hope that a solution may be found. Those may be “the push and the pull” or “the stick and the carrot” mighty forces that prompt more and more scientists and lay persons to move over to the camp of “treating aging as a disease,” toward investing more and more time and effort for its amelioration or even cure, as soon as possible, for the benefit of all. Yet, the very idea of “treating aging as a disease,” or some other title given to a morbid, debilitating and deadly condition, is by no means an intellectual novelty. It is a long established commonsensical intellectual tradition and a profound and ancient human desire. Yet, with the growing aging population and increasing technological capabilities, this idea is achieving an ever greater prominence. Eventually, the question whether aging should be considered “a treatable disease” may be reduced to technological capacity and semantics. While degenerative aging, that is the accumulation of structural damage, impairment of metabolic balance and functioning, may be seen as a disabling and deteriorative process that requires prevention and treatment, using advanced biomedical technology; the achievement of healthy longevity may be its cure.

## Conflict of interest statement

The author declares that the research was conducted in the absence of any commercial or financial relationships that could be construed as a potential conflict of interest.
